# Supplementary material for: Transglutaminases Are Active in Perivascular Adipose Tissue
Source: Int J Mol Sci. 2021 Mar 5;22(5):2649. doi: 10.3390/ijms22052649 (PMC7961980; doi:10.3390/ijms22052649)
Supplement: Supplementary file 1 [file ijms-22-02649-s001.pdf]

Supplementary material

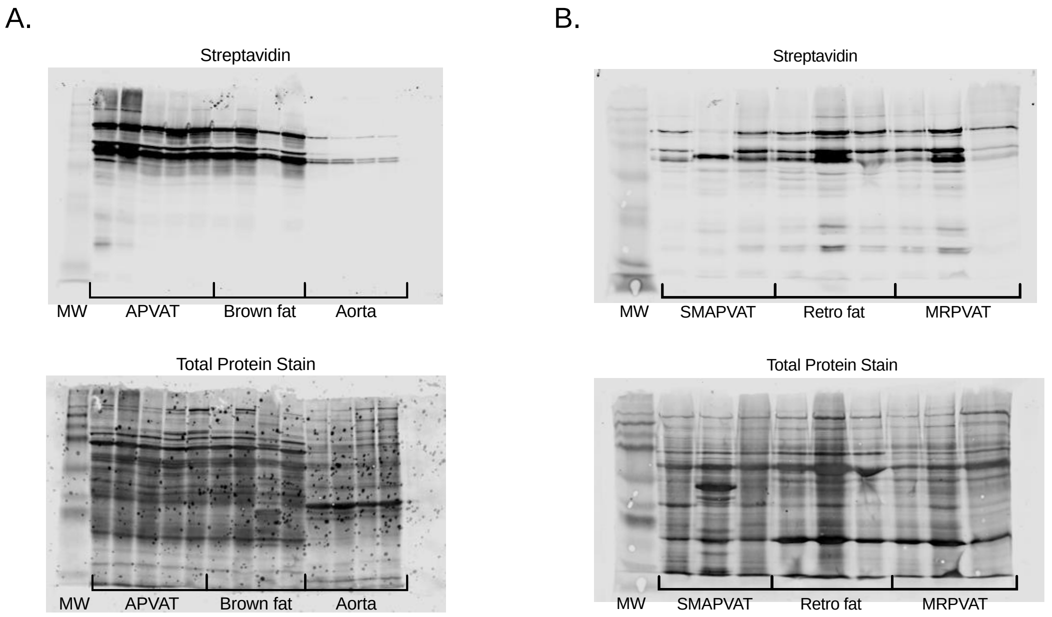

Figure S1. Western blots depicting endogenous biotin content in APVAT, Brown fat, and aorta (**A** top) and SMAPVAT, retroperitoneal fat, and MRPVAT (**B** top), and their respective total protein stains (**A**,**B** bottom). $n \geq 3$ for each tissue type.
